# HEALTH OUTCOMES OF AGING INDIVIDUALS EXPOSED TO HARMFUL ALGAL BLOOMS IN SOUTH FLORIDA

**DOI:** 10.1093/geroni/igae098.0859

**Published:** 2024-12-31

**Authors:** Rebecca Koszalinski, Malcolm McFarland, Michael Parsons, John Reif, Adam Schaefer, Judyta Kociolek

**Affiliations:** University of Central Florida, Greenacres, Florida, United States; Florida Atlantic University, Boca Raton, Florida, United States; Florida Gulf Coast University, Fort Myers, Florida, United States; Colorado State University, Fort Collins, Colorado, United States; Abt Associates Inc, Richmond Hill, Georgia, United States; Florida Atlantic University, Boca Raton, Florida, United States

## Abstract

Environmental changes have profoundly altered the lives of individuals in South Florida. Harmful Algal Blooms (HAB) and associated toxins produced by cyanobacteria and Karenia brevis impact the lives of Floridians, including aging individuals who recreate on the water in their retirement years. This interprofessional team of scientists and health professionals is concluding Year-5 of a longitudinal study to learn short-and long-term health impacts. Participants were recruited from publicly accessible sites, community groups, social media posts and in person events (Schaefer et al., 2020). Participants completed a questionnaire includes location, type, and duration of recreational or occupational contact with surface water and local beaches within the last 10 days. Activities included swimming, wading, boating, kayaking, paddle-boarding, tubing, fishing, and visiting a beach. Participants reported recreational fishing activities and consumption of locally caught seafood from impacted water bodies. Reif, et al. (2023) indicated that the most commonly reported symptoms were rhinorrhea, sneezing, headache, sore throat and dry cough. There was an increase of respiratory symptoms (74% of respondents), ocular symptoms (62% of respondents), and gastrointestinal symptoms (35% of respondents). Residential exposure was significantly associated with increased reporting of dry cough (p = 0.03), dyspnea (p < 0.01) and wheezy respirations (p = 0.04). Among persons reporting gastrointestinal symptoms, nausea (p = 0.02) and abdominal pain (p < 0.01) were significantly associated with residential exposure. Recreational exposure was significantly associated with sore throat and eye irritation. A secondary data analysis will specifically investigate the health impacts of aging individuals in this dataset.

